# Gait Characteristics Harvested during a Smartphone-Based Self-Administered 2-Minute Walk Test in People with Multiple Sclerosis: Test-Retest Reliability and Minimum Detectable Change

**DOI:** 10.3390/s20205906

**Published:** 2020-10-19

**Authors:** Alan K. Bourke, Alf Scotland, Florian Lipsmeier, Christian Gossens, Michael Lindemann

**Affiliations:** 1 Roche Pharma Research and Early Development, pRED Informatics, Roche Innovation Center Basel, F Hoffmann–La Roche Ltd., Grenzacherstrasse 124, 4070 Basel, Switzerland; alan.bourke@roche.com (A.K.B.); florian.lipsmeier@roche.com (F.L.); michael.lindemann@roche.com (M.L.); 2 Inovigate, Aeschenvorstadt 55, 4051 Basel, Switzerland; alf.scotland@inovigate.com

**Keywords:** gait, smartphone, multiple sclerosis, 2-minute walk test, test-retest reliability, MDC, SEM, wearable sensors

## Abstract

The measurement of gait characteristics during a self-administered 2-minute walk test (2MWT), in persons with multiple sclerosis (PwMS), using a single body-worn device, has the potential to provide high-density longitudinal information on disease progression, beyond what is currently measured in the clinician-administered 2MWT. The purpose of this study is to determine the test-retest reliability, standard error of measurement (SEM) and minimum detectable change (MDC) of features calculated on gait characteristics, harvested during a self-administered 2MWT in a home environment, in 51 PwMS and 11 healthy control (HC) subjects over 24 weeks, using a single waist-worn inertial sensor-based smartphone. Excellent, or good to excellent test-retest reliability were observed in 58 of the 92 temporal, spatial and spatiotemporal gait features in PwMS. However, these were less reliable for HCs. Low SEM% and MDC% values were observed for most of the distribution measures for all gait characteristics for PwMS and HCs. This study demonstrates the inter-session test-retest reliability and provides an indication of clinically important change estimates, for interpreting the outcomes of gait characteristics measured using a body-worn smartphone, during a self-administered 2MWT. This system thus provides a reliable measure of gait characteristics in PwMS, supporting its application for the longitudinal assessment of gait deficits in this population.

## 1. Introduction

Multiple sclerosis (MS) is a chronic autoimmune inflammatory disease of the central nervous system [1], which can gradually lead to gait deficits and the inability to fully activate the muscles of the lower limbs [2]. It has been shown that gait impairment affects quality of life, health status and productivity [3] in persons with MS (PwMS), with the prevalence of these reported impairments between 75% and 90% [4]. Analysis of gait, therefore, plays a central role in the assessment of MS disease severity and progression. 

MS diagnosis is based on established clinical and magnetic resonance imaging (MRI) criteria [5] yet to date there is no universal gold standard clinical assessment for MS disease severity [6]. Current best practice includes administering of observational rating scales, such as the expanded disability status scale (EDSS) and the multiple sclerosis impact scale (MSIS-29), and ma+nually timed functional tasks (e.g., timed-up-and-go (TUG), timed 25-foot walk (T25FW) and the 2- or 6-minute walk test (2MWT, 6MWT)) administered by trained clinical staff [7]. Central to these clinical assessments is the quantification of gait deficits and gait characteristics in PwMS. However these assessments are administered infrequently, can be time- and resource-intensive and are prone to rater-dependent error. Thus a quantitative assessment of gait using body-worn inertial-sensor based systems has the potential to provide objective longitudinal monitoring of the disease state in PwMS.

The 2-minute walk test is a validated alternative to the 6-minute walk test [8]. The distance travelled in a fixed amount of time is used as a measure of physical function [8]. An opportunity thus exists as the participant’s gait characteristics remain unexplored. Recent studies have employed body-worn inertial-sensor based systems for the assessment of gait in PwMS, through the measurement of temporal, spatial and/or spatiotemporal gait characteristics, during laboratory-based scripted functional assessments such as the 6MWT [9,10,11], T25FW [12,13], TUG [13,14,15], a 1-minute walk [16] and free-living gait [16]. However these studies suffer from a number of limitations and drawbacks, including the requirement for a multi-sensor body-worn set-up [9,11,12,13,14,15,17], the test requires assistance from a clinician and/or were laboratory based [9,11,12,13,14,15,17], were carried out on a small/limited cohort [10,16] or the sensor requires direct attachment to the skin [9,14]. 

**Figure 1:**
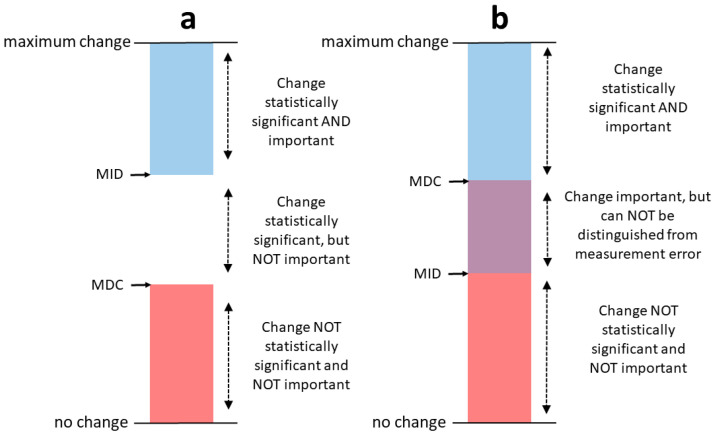
In (**a**) the MID is larger than the MDC. In this situation, changes as large as the MID can be considered statistically significant and important to patients. In (**b**) the MID is smaller than the MDC. In this situation, changes as large as the MID may be important for patients, but they cannot be distinguished from measurement error [19]. Minimal detectable change (MDC). Minimal important difference (MID). Figure adapted from Terwee et al. [20].

Advances in inertial-sensor technology combined with the widespread adoption of commercially available smartphones, has made the ubiquitous monitoring of human body movement, using body-worn inertial-sensors, more possible than ever before. To date, to the best of our knowledge, no study has demonstrated that temporal, spatial and spatiotemporal gait characteristics can be successfully measured using a single sensor at the waist in an MS cohort, during an unsupervised, self-administered test. We thus hypothesize that instrumentation of the 2MWT performed in the person’s home environment, with a body-worn inertial-sensor based smartphone, can potentially reveal gait characteristics related to MS disease state, without a supervising clinician or a laboratory. Identifying when a patient’s behavior has changed due to an intervention, through the use of body-worn sensors, has the potential to expand the armamentarium beyond the traditional clinical endpoints. Interpreting this change requires two benchmarks: the minimum detectable change (MDC), and the minimal important difference (MID). The MDC is the minimum magnitude of change required to be 95% confident that the observed change between measurements reflects true change and not measurement error [18]. The MID is defined as the smallest change in the score of the construct to be measured, which is perceived as important by patients, clinicians, or relevant others [19]. If the MDC is smaller than the MID, it is possible to distinguish a clinically important change from measurement error with a large amount of certainty [20], Figure 1. However, this is much more difficult if the MDC is larger than the MID, since there is a considerable chance that the observed change is caused by measurement error [20], Figure 1. 

MDC is thus a prerequisite that allows clinicians and researchers to explain change scores in successive measurements reasonably [21]. The objective was thus to assess the test-retest reliability and ultimately the MDC of gait characteristics measured during a self-administered 2MWT with a body–worn inertial-sensor based smartphone, in PwMS and healthy controls (HCs). 

## 2. Materials and Methods

Extensive details on the trial protocol, inclusion/exclusion criteria and ethical approval are available in the study by Midaglia et al. [22] with the key relevant points presented here.

### 2.1. Study Design

This cross-sectional study examined the longitudinal inter-session test-retest reliability and MDC of gait characteristics harvested during the self-administered 2MWT in PwMS and HCs. 

### 2.2. Participants

In total, 76 PwMS and 25 HCs were enrolled in the study. PwMS were included if they had a diagnosis of MS and an EDSS score of 0.0 to 5.5 (inclusive). The EDSS [23] is a 10-point ordinal scale used to measure MS disability. Zero indicates normal neurological exam, while higher values indicate greater disability and increased mobility impairment.

### 2.3. Protocol and Equipment

**Figure 2:**
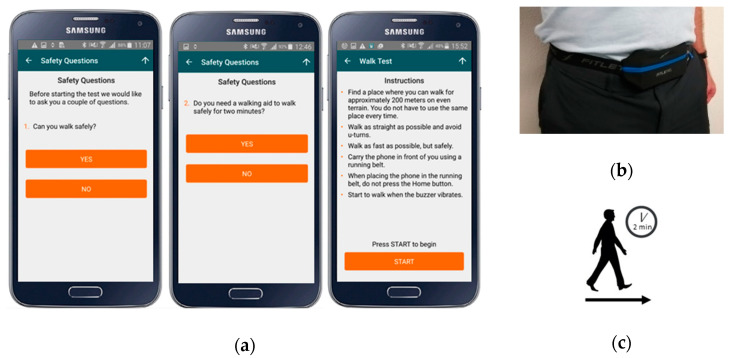
(**a**) The instructional screens provided to the participant, (**b**) a smartphone attached at the waist using a belt-bag and (**c**) summary description of the 2-minute walk test (2MWT).

At enrolment/baseline, PwMS and HCs were provided with a preconfigured smartphone and were asked to perform the 2MWT daily over a 24-week period. For each test, participants are first presented with safety questions, given instructions how to perform the test and how to attach the smartphone, Figure 2. Participants then record the test by attaching the smartphone at the front of the body in a commercial waist-worn belt-bag or in a trouser pocket, Figure 2. Audio and vibration cues indicate the beginning and end of the 2-minute test. Feedback on how participants perform the test is not provided.

**Figure 3:**
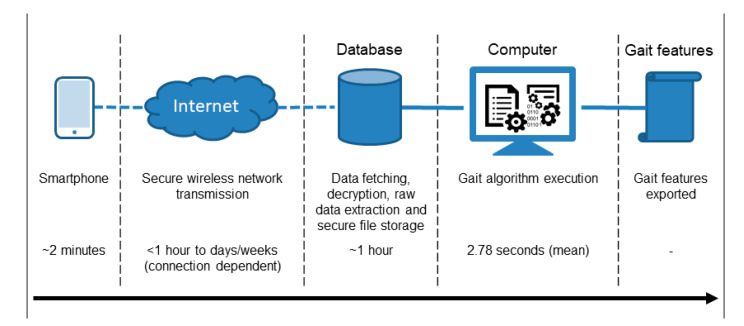
Data flow and approximate timeline which includes performing the 2MWT, wireless transmission of inertial monitoring unit (IMU) data, database processing and file extraction, gait algorithm execution and gait feature export.

Participants were provided with a Samsung Galaxy S7 smartphone by the study investigator. The Samsung Galaxy S7 contains the LSM6DS3 from STMicroelectronics. The LSM6DS3 is a micro-electro-mechanical systems inertial monitoring unit (IMU) that contains factory calibrated tri-axial accelerometer and gyroscope sensors. The Samsung Galaxy S7 samples used a variable sampling rate of approximately 100 Hz. The smartphone’s sensor data were encrypted before wireless transmission to a secure database for subsequent data analysis before gait features were exported, Figure 3.

### 2.4. Signal Processing

Data analysis was performed in Python 3.6. The data harvested during each 2MWT were first resampled using a Blackman window to create a regular sampling rate, then filtered using a zero-phase 2nd order Butterworth filter [24] at a cut-off frequency of 25 Hz. The IMU’s longitudinal axis was aligned with the gravity vector using an axis-angle rotation [25]. The gravity vector was estimated using the mean of the accelerometer data during the walking bouts.

### 2.5. Phone Location Detection

The location of the smartphone was inferred based on the smartphone orientation during each 2MWT. If a landscape orientation was detected, a waist worn location was assumed.

### 2.6. Walking Bout Algorithm

Walking bouts were identified through thresholding of the standard-deviation of the vector-magnitude of the tri-axial accelerometer signals, using a non-overlapping rolling window of 1-second. Walking was detected if the signal exceeded a threshold of 1.0.

### 2.7. Gait Algorithms

**Figure 4:**
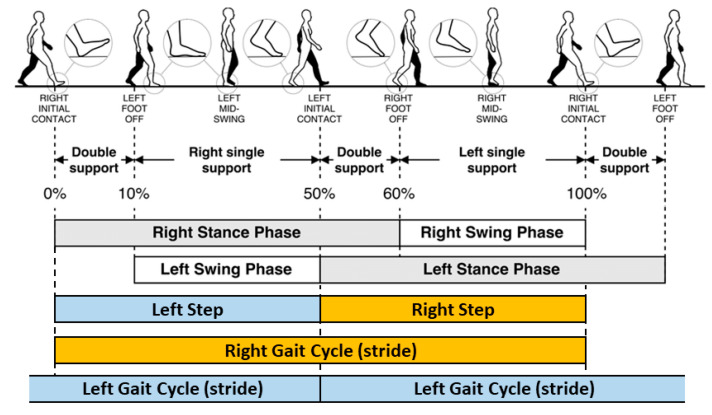
Schematic of the human gait cycle and a selection of the temporal gait characteristics measured in this study. Reproduced and modified with permission from Tunca, C.; Pehlivan, N.; Ak, N.; Arnrich, B.; Salur, G.; Ersoy, C. Inertial Sensor-Based Robust Gait Analysis in Non-Hospital Settings for Neurological Disorders. *Sensors* **2017**, *17*, 825.

The following gait characteristics were measured for each 2MWT recorded only at the waist (trouser pocket location excluded); step/stride length [26], step/stride velocity [27], stance time [26], swing time [26] and step/stride time [26], Figure 4.

The calculation of these gait characteristics is extensively described in [27], along with a software implementation tutorial [28], but summarized here for convenience.

**Figure 5:**
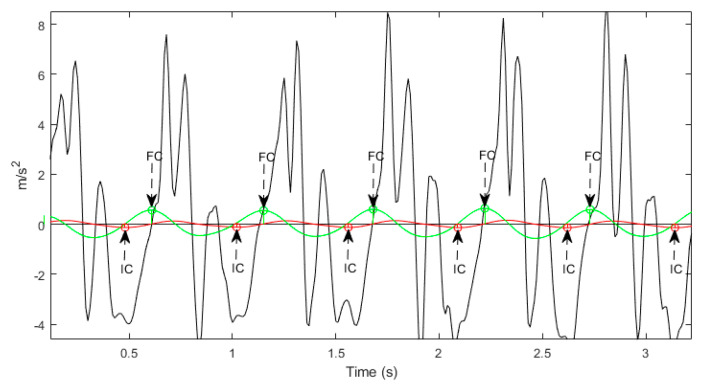
The heel-contact/initial contact (IC) and toe-off/final contact (FC) points identified from local minima and local maxima of the Gaussian continuous wavelet transform, of the processed vertical accelerometer signal.

During the gait cycle, the initial contact (IC, heel strike) and final contact (FC, foot-off) events, were identified from the Gaussian continuous wavelet transform of the vertical accelerometer signal, Figure 5. The detected IC and FC events were used to compute estimates of step, stance, swing and stride times [29]. The IC events were also used to provide an estimate of step length using the inverted pendulum model [27] along with the scaled iliac height of each subject. Step velocity was calculated as the ratio of step length to step time [27]. Validation of these algorithms [27] was performed in both young and older adult populations [27] and in Parkinson’s disease patients [30]. Stride length was the sum of consecutive contralateral step lengths, with stride velocity the ratio of stride length to stride time. The definitions of the computed gait characteristics are presented in Table 1. 

Inertial sensor gait data were segmented into straight line walking bouts, of minimum 10 s, with turns greater than 45^o^ excluded. If multiple gait bouts were detected, gait characteristics computed for each walking bout were compiled for each 2MWT. 

The following features were computed for each gait characteristic during each 2MWT. To calculate variability, the variance for all steps/strides (left and right separately) was calculated and combined [27]. Left and right steps were determined using the anterior-posterior gyroscope signal [29]. The standard deviation (std) was calculated using all steps/strides regardless of side. The coefficient-of-variation (CV) [31] was calculated using Equation (1):

CV = std/mean(1)

Asymmetry was determined as the absolute difference between left and right steps [27]. The following distribution measures were calculated for each 2MWT. The; 5th, 25th, 50th, 75th and 95th percentiles, referred to from here on as percentiles, as well as the maximum, mean and minimum. The inclusion of percentile calculations is motivated by a recent finding, where the 95th percentile of stride velocity was considered by regulators as a new endpoint when assessing a neurological disease [32]. 

### 2.8. Gait Feature Aggregation

The median of each gait feature, calculated during individual 2MWTs, was harvested during a 14-day period of tests, with a minimum of three tests required. This constituted one session and was compiled for each participant. A 14-day period was chosen to reduce day-to-day/weekend-weekday fluctuations and variations in the daily test schedule/location which are all irrelevant for longer MS disease progression.

### 2.9. Test-Retest Reliability

Test-retest reliability was assessed using the second (baseline session excluded to allow familiarisation) and subsequent complete 14-day session per subject. This was performed using a two-way mixed effect model with absolute agreement for a single rater/measurement intra-class correlation coefficient, ICC (2,1) [33]. Reliability is indicated using the ICC values classified as; poor (ICC < 0.5), moderate (ICC = 0.5 to 0.75), good (ICC = 0.75 to 0.9) and excellent (ICC > 0.9) [34].

### 2.10. Standard Error of Measurement (SEM) and Minimum Detectable Change (MDC)

The standard error of measurement (SEM) reflects the precision of the measurement instrument [34]. The SEM was calculated as Equation (2):

(2) $$SEM=SD\cdot \sqrt{1-ICC}$$

where SD is the standard deviation of the specific gait characteristic values. The minimal detectable change with a 95% confidence interval (MDC) was calculated to determine how much measured change is likely to reflect true change [34]. The MDC [18] was calculated as Equation (3):

(3) $$MDC=1.96\cdot SEM\cdot \sqrt{2}$$

The SEM and MDC can be expressed as percentages that are independent of the units of measurement. This allows comparison on the amount of random error between measurement features, as Equation (4):

(4) $$SEM\%=100\cdot (SEM/\overset{-}{x}),$$

and as Equation (5):

(5) $$MDC\%=100\cdot (MDC/\overset{-}{x}),$$

where $\overset{-}{x}$ is the mean for all observations [18]. SEM% values were classified as low (SEM% ≤ 10%) or high (SEM% > 10%). MDC% values were classified as low (MDC% ≤ 20%), acceptable (20% < MDC% < 40%) and high (MDC% ≥ 40%). Thresholds for SEM% and MDC% were chosen to align with previous similar work [35] as no clear criteria for SEM% and MDC% are available.

## 3. Results

A total of 4854 tests were recorded from the waist (trouser pocket location excluded from this analysis) from 101 participants. Baseline demographics are described in Table 2.

There was no significant difference (*p* = 0.569) between the quantity of 2MWTs performed during each 14-day session by PwMS (n = 3867, 6.66 ± 4.6, maximum = 17, minimum = 1) and HCs (n = 987, 6.49 ± 4.6, maximum = 15, minimum = 1) and no significant difference (*p* = 0.235) between the total walking bout length during each 2MWT performed by PwMS (n = 3867, 92.69 s ± 36.60 s) and HCs (n = 987, 91.9 s ± 36.6 s). Algorithm execution computation time (Figure 3) was 3 h 45 min to process 4854 recorded 2MWT files (mean 2.78 s per file) using a HP computer with an Intel® Core^tm^ i5-8350U with a CPU rate of 1.70 GHz and 16 GB of RAM.

### 3.1. Test-Retest Reliability

Data from a total of 62 participants (51 PwMS and 11 HCs) were included to assess test-retest reliability, presented in Table 3 and Table 4 for PwMS and HCs. 

For PwMS, of the 48 temporal gait features, 2 were excellent (ICC > 0.9), 31 were good to excellent (ICC > 0.75), 3 were moderate to excellent and 11 were moderate to good, Table 3. Of the 44 spatial and spatiotemporal gait features, 12 were excellent (ICC > 0.9), 13 were good to excellent (ICC > 0.75), 4 were moderate to excellent, 12 were moderate to good, and 3 were poor to good, Table 4.

For HCs, of the 48 temporal gait features, 1 was excellent (ICC > 0.9), 16 were good to excellent (ICC > 0.75), 21 were moderate to excellent, 3 were poor to good and 17 were poor to excellent, Table 3. Of the 44 spatial and spatiotemporal gait features, 12 were good to excellent (ICC > 0.75), 11 were moderate to excellent, 8 poor to excellent and 13 were poor to good, Table 4.

### 3.2. SEM and MDC

For PwMS, Table 4 for the percentiles, min, mean and max of spatial (step and stride length) and spatiotemporal (step and stride velocity) gait characteristics, the SEM and SEM% values were low (4.16−8.94%) except for min which produced high values and in some cases max and the 5th percentile of step length and velocity. The MDC and MDC% values were low or acceptable (11.53−37.28%) except for some cases of min and max which were high. The std, CV, variability and asymmetry produced high SEM and SEM% values (19.18−53.39%) and high MDC and MDC% values (53.15−148%). For the percentiles, min, mean and max of temporal gait characteristics (stance, step, swing and stride time) the SEM and SEM% values were low (2.35−9.43%), except for the min of step and swing time (12.41%, 13.24%), Table 3. MDC and MDC% values were low or acceptable (6.51−36.71%). The std, CV, variability and asymmetry produced high SEM, SEM% (21.42−67.33%), MDC and MDC% values (59.37−186.63%).

For HCs, Table 4 for the percentiles, min, mean and max of spatial and spatiotemporal gait characteristics the SEM and SEM% values were low (2.49−8.22%) except for min which produced high values and in some cases max and the 5th percentile of step length and velocity. The MDC and MDC% values were low or acceptable (6.89−28.45%) except for the min and 5th percentile of step length and velocity which were high. The std, CV, variability and asymmetry produced high SEM, and SEM% values (23.51−59.55%) and high MDC and MDC% values (65.18−165.07%). For HCs for the percentiles, min, mean and max of temporal gait characteristics (stance, step, swing and stride time) the SEM and SEM% values were low (1.53−6.59%) and the MDC and MDC% values were also low or acceptable (4.23−24.83%), Table 3. The std, CV, variability and asymmetry produced high SEM, and SEM% values (13.66−46.96%) and acceptable or high MDC and MDC% values (37.86−130.18%).

## 4. Discussion

This study evaluated the between-session test-retest reliability, SEM and MDC of a comprehensive set of gait features harvested during a self-administered 2-minute walk test, using a single smartphone attached to the front of the waist in PwMS and HCs. 

ICC values for test-retest reliability were similar to those reported in other assessments of gait in PwMS using body-worn IMUs [11,15] and for clinical walking outcome assessments in PwMS [36]. ICCs for test-retest reliability harvested from HCs were less reliable than from PwMS. Analysis of the ICC over 4 consecutive pairs of sessions (compliance dropped below 50% after the initial 8 sessions) confirmed that the test-retest robustness estimates were unaffected by the choice of session for PwMS. For HCs we observed some instability due to the smaller sample size, which is also reflected in the larger confidence intervals reported. Bootstrap analysis with 1000 iterations on the study participants confirmed that the results reported here are robust to the choice of participants. Koo and Li et al. [33] recommend that “researchers should try to obtain at least 30 heterogeneous samples” when conducting a reliability study. In this study we harvested values from 51 PwMS and 11 HCs thus the quantity of values recorded from HCs is much less than recommended.

The SEM was used to assess the measurement precision of the gait features and the MDC to detect the minimal threshold of change above the 95% confidence interval (CI) for each feature. These measures represent the limit for the smallest change that indicates a real or detectable improvement rather than measurement error for people with MS, thus, making it particularly useful for gauging the effects of an intervention or rehabilitation [18].

Small or low SEM values for gait characteristics indicate that measurements are stable and reproducible over time, thus indicating the precision of the measurement system [37]. Angelini et al. [11] reported SEM and MDC values for gait characteristics measured in PwMS (n = 26, EDSS = 2–6.5) using three body-worn (both ankles and lumbar spine) IMU-based sensors, during a 6MWT recorded in a clinical setting. SEM values reported here are lower for mean stride (0.026 s vs. 0.04 s), step (0.013 s vs. 0.02 s) and stance (0.017 s vs. 0.03 s) times, Table 3, and comparable to the swing time (0.012 s vs. 0.01 s), Table 3, reported by Angelini et al. [11]. MDC values reported here are lower for step (0.036 s vs. 0.05 s) time and higher for stride (0.072 s vs. 0.01 s), stance (0.046 s vs. 0.03 s) and swing (0.032 s vs. 0.03 s) time, Table 3, to those reported by Angelini et al. [11].Except for swing time asymmetry, higher SEM and MDC temporal variability and asymmetry metrics were observed for mean stride, step, stance and swing times when compared to those reported by Angelini et al. [11]. The study by Angelini et al. [11] was however conducted in a controlled clinical setting using a multi-sensor recording set-up. Future work will examine the quantity of steps/strides required to produce more reliable measures of gait, especially for variability and asymmetry metrics, similar to Hollman et al. [38].

Learmonth et al. [36] demonstrated that in an MS population (n = 82, SR-EDSS = 0–6.5) the 6MWT has an MDC% of 20% for both distance and speed. Examining MS subgroups this changes to 11% and 39% for MS mild (SR-EDSS < 4) and MS moderate (SR-EDSS = 4–6.5), respectively, for both the distance and speed. Valet et al. [39] reported an 11% MDC% for the 2MWT in an MS population (n = 63, EDSS = 0–4). In this study for the MDC% of the percentiles, min, mean, max of the spatial, spatiotemporal and temporal gait features, 46 of the 64 features (Table 3 and Table 4) were below the 20% MDC% reported by Learmonth et al. [36] with 13 of the 64 below the 11% MDC% reported by Valet et al. [39]. For the MDC% for variability, std, CV and asymmetry none were less than 39%.

Decavel et al. [40] reported SEM% and MDC% values for gait characteristics harvested during the T25FW using a GAITRite® (CIR Systems Inc. Franklin, NJ, USA) sensorised walkway in a population of 59 PwMS (EDSS mean (SD) of 5.2 (1.1)) and 19 HCs. Here, we report lower SEM% and MDC% values for mean stride length and velocity (gait velocity versus stride/step velocity) for both PwMS and HCs and lower values for stride time for PwMS, Table 4. Thus demonstrating that the measurement of gait characteristics can be achieved in a person’s own home environment, using an unsupervised self-administered walking test, using a single sensor attached to the waist and can achieve better precision and lower measurement error than a dedicated laboratory based system.

The obtained SEM and MDC values can be considered as valid measurement threshold references for their respective gait characteristics when measured using a single body-worn smartphone, during an unsupervised self-administered 2MWT, performed in the person’s home environment. Future further studies in this domain should examine the, clinically relevant, MID for outcomes of these gait characteristics, as they are currently lacking. Knowledge of these MID values would assist in the clinical interpretation of the indices for the measurement errors found in this study. This will help clinicians and researchers reasonably and confidently determine real change, between repeated session measurements, for PwMS evaluated using a body-worn smartphone during a self-administered 2MWT.

Future work will determine the accuracy of this algorithm using ground truth data harvested from an MS cohort. Future work will also examine the criterion validity of these gait characteristics, measured in an MS cohort, with reported outcomes from clinical observational rating scales (e.g., EDSS and MSIS-29) and timed functional assessments (e.g., T25FW). In addition, variations in inertial sensor data measured using different smartphones do exist [41], future work will employ techniques such as auto-calibration [42] to reduce the error associated with these variations.

## 5. Conclusions

These findings indicate that gait analysis performed on inertial sensor data harvested during a self-administered 2MWT using a single smartphone, attached to the front of the waist, provides both accurate and reliable measurements of selected spatial, temporal and spatiotemporal characteristics of gait in PwMS and HCs. 

The ICC, SEM and MDC values recorded for this measurement system are also comparable to existing clinically accepted outcome measures for gait evaluation. 

These findings have meaningful implications for clinicians and researchers who use body-worn IMU-based measurement systems to evaluate gait deficits in PwMS.

## Figures and Tables

**Table 1:** Definitions of gait characteristics, reprinted from Hollman, J.H.; McDade, E.M.; Petersen, R.C. Normative Spatiotemporal Gait Parameters in Older Adults. *Gait Posture* **2001**, *34*, 111–118, Copyright (2011), with permission from Elsevier.

Name	Description
***Spatial***	
**Step Length**	The anterior-posterior distance between the heel of one footprint to the heel of the opposite footprint
**Stride Length**	Anterior-posterior distance between heels of two consecutive footprints of the same foot (left to left, right to right); two steps (e.g., a right step followed by a left step) comprise one stride or one gait cycle
***Spatiotemporal***	
**Step Velocity**	Calculated by dividing the step length by the step time
**Stride Velocity**	Calculated by dividing stride length by the stride time
***Temporal***	
**Stance Time**	The stance phase is the weight bearing portion of each gait cycle initiated at heel contact and ending at toe off of the same foot; stance time is the time elapsed between the initial contact and the last contact of a single footfall
**Step Time**	Time elapsed from initial contact of one foot to initial contact of the opposite foot
**Stride Time**	Time elapsed between the initial contacts of two consecutive footfalls of the same foot
**Swing Time**	The swing phase is initiated with toe off and ends with initial contact of the same foot; swing time is the time elapsed between the last contact of the current footfall to the initial contact of the next footfall of the same foot

**Table 2:** Cohort demographics and characteristics for PwMS and HCs at baseline. Abbreviations: EDSS: Expanded Disability Status Scale, HC: Healthy control, MS: Multiple sclerosis, NA: Not applicable, PPMS: Primary progressive multiple sclerosis, PwMS: People with multiple sclerosis, RRMS: Relapsing-remitting multiple sclerosis, SD: Standard deviation, SPMS: Secondary progressive multiple sclerosis, T25FW: Timed 25-Foot Walk.

Parameter	PwMS	HCs
Subjects (n)	n = 76	n = 25
Age, mean ± SD, years	39.5 ± 7.9	34.9 ± 9.3
Female, *n* (%)	53 (69.7)	7 (28.0)
MS diagnosis (PPMS, SPMS, RRMS), %	3.9, 5.3, 90.8	NA
Time since MS symptom onset, mean ± SD, years	11.3 ± 7.0	NA
EDSS, mean ± SD	2.4 ± 1.4	NA
T25FW, mean ± SD, seconds	6.0 ± 2.1	5.0 ± 1.0

**Table 3:** Reliability statistics for temporal gait characteristics for PwMS and HCs. Colour code- **ICC**: Poor < 0.5, moderate = 0.5–0.75, good = 0.75–0.9, excellent > 0.9; **SEM%**: low ≤10%, High > 10%. **MDC%**: low ≤20%, 20% < acceptable < 40%, high ≥40%.

PwMS									HCs								
	ICC (2,1)	(lb,	ub)	p	SEM	SEM (%)	MDC	MDC (%)		ICC (2,1)	(lb,	ub)	p	SEM	SEM (%)	MDC	MDC (%)
Temporal									Temporal								
**stance time (s)**									**stance time (s)**								
0.05	0.83	0.72	0.90	<0.001	0.034	5.87	0.094	16.27	0.05	0.80	0.27	0.95	<0.001	0.020	3.27	0.056	9.07
0.25	0.81	0.69	0.89	<0.001	0.028	4.49	0.078	12.43	0.25	0.86	0.59	0.96	<0.001	0.017	2.54	0.046	7.05
0.5	0.88	0.80	0.93	<0.001	0.023	3.46	0.063	9.59	0.5	0.67	0.20	0.90	0.006	0.030	4.53	0.084	12.55
0.75	0.91	0.84	0.95	<0.001	0.025	3.60	0.069	9.97	0.75	0.79	0.42	0.94	0.001	0.029	4.12	0.079	11.43
0.95	0.90	0.83	0.94	<0.001	0.037	4.96	0.102	13.76	0.95	0.91	0.71	0.97	<0.001	0.027	3.68	0.075	10.19
max	0.82	0.70	0.89	<0.001	0.060	7.45	0.167	20.65	Max	0.92	0.74	0.98	<0.001	0.027	3.46	0.076	9.58
mean	0.92	0.87	0.95	<0.001	0.017	2.54	0.046	7.05	Mean	0.77	0.35	0.93	0.001	0.025	3.66	0.068	10.15
min	0.73	0.56	0.83	<0.001	0.047	9.22	0.131	25.57	Min	0.89	0.63	0.97	<0.001	0.028	5.33	0.078	14.76
std	0.87	0.79	0.93	<0.001	0.018	33.95	0.051	94.11	Std	0.89	0.63	0.97	<0.001	0.009	21.82	0.026	60.49
CV	0.86	0.76	0.92	<0.001	0.029	34.96	0.079	96.90	CV	0.88	0.56	0.97	<0.001	0.013	20.75	0.035	57.53
variability	0.85	0.75	0.91	<0.001	0.019	38.41	0.053	106.46	variability	0.85	0.52	0.96	<0.001	0.009	23.70	0.026	65.70
asymmetry	0.86	0.76	0.92	<0.001	0.013	48.19	0.035	133.57	asymmetry	0.89	0.66	0.97	<0.001	0.011	46.96	0.029	130.18
**step time (s)**									**step time (s)**								
0.05	0.86	0.77	0.92	<0.001	0.030	6.81	0.084	18.87	0.05	0.59	0.08	0.87	0.013	0.032	6.59	0.088	18.25
0.25	0.74	0.59	0.84	<0.001	0.031	6.35	0.085	17.59	0.25	0.92	0.74	0.98	<0.001	0.010	2.03	0.029	5.63
0.5	0.89	0.81	0.93	<0.001	0.016	3.10	0.045	8.59	0.5	0.89	0.65	0.97	<0.001	0.013	2.43	0.036	6.74
0.75	0.95	0.91	0.97	<0.001	0.013	2.35	0.036	6.51	0.75	0.81	0.47	0.95	0.001	0.023	4.13	0.065	11.44
0.95	0.92	0.87	0.96	<0.001	0.025	4.18	0.070	11.59	0.95	0.98	0.94	1.00	<0.001	0.009	1.53	0.025	4.23
max	0.85	0.75	0.91	<0.001	0.046	6.90	0.128	19.13	max	0.87	0.59	0.96	<0.001	0.031	4.73	0.086	13.11
mean	0.91	0.85	0.95	<0.001	0.013	2.49	0.036	6.89	mean	0.80	0.43	0.94	0.001	0.017	3.25	0.048	9.00
min	0.69	0.51	0.81	<0.001	0.047	12.41	0.129	34.39	min	0.89	0.61	0.97	<0.001	0.029	7.54	0.081	20.89
std	0.90	0.83	0.94	<0.001	0.015	28.27	0.043	78.35	std	0.96	0.86	0.99	<0.001	0.006	13.94	0.017	38.65
CV	0.89	0.81	0.94	<0.001	0.030	28.83	0.083	79.90	CV	0.96	0.85	0.99	<0.001	0.011	13.66	0.029	37.86
variability	0.88	0.81	0.93	<0.001	0.015	32.04	0.043	88.82	variability	0.94	0.81	0.98	<0.001	0.005	14.01	0.015	38.84
asymmetry	0.70	0.52	0.81	<0.001	0.018	55.74	0.050	154.50	asymmetry	0.91	0.72	0.98	<0.001	0.012	37.13	0.033	102.92
**stride time (s)**									**stride time (s)**								
0.05	0.86	0.76	0.91	<0.001	0.048	5.10	0.133	14.13	0.05	0.57	0.05	0.86	0.014	0.053	5.32	0.148	14.74
0.25	0.75	0.60	0.85	<0.001	0.050	4.93	0.137	13.66	0.25	0.79	0.42	0.94	0.001	0.035	3.26	0.096	9.05
0.5	0.90	0.83	0.94	<0.001	0.030	2.88	0.083	7.97	0.5	0.82	0.48	0.95	<0.001	0.035	3.21	0.096	8.90
0.75	0.94	0.89	0.96	<0.001	0.027	2.49	0.075	6.91	0.75	0.84	0.54	0.96	<0.001	0.033	3.03	0.092	8.39
0.95	0.92	0.87	0.95	<0.001	0.039	3.43	0.108	9.50	0.95	0.93	0.77	0.98	<0.001	0.030	2.56	0.082	7.11
max	0.79	0.66	0.87	<0.001	0.082	6.73	0.229	18.67	max	0.87	0.60	0.96	<0.001	0.045	3.67	0.124	10.16
mean	0.91	0.86	0.95	<0.001	0.026	2.48	0.072	6.88	mean	0.78	0.39	0.94	0.001	0.036	3.37	0.101	9.34
min	0.68	0.50	0.80	<0.001	0.080	9.43	0.222	26.15	min	0.92	0.70	0.98	<0.001	0.043	4.92	0.119	13.64
std	0.87	0.78	0.92	<0.001	0.023	35.44	0.064	98.24	std	0.74	0.25	0.93	0.001	0.016	29.91	0.044	82.92
CV	0.84	0.74	0.91	<0.001	0.025	39.06	0.069	108.27	CV	0.73	0.22	0.92	0.001	0.014	29.26	0.039	81.12
variability	0.87	0.78	0.92	<0.001	0.022	35.13	0.062	97.37	variability	0.75	0.25	0.93	0.001	0.016	29.51	0.043	81.80
asymmetry	0.94	0.90	0.97	<0.001	0.006	67.33	0.017	186.63	asymmetry	0.75	0.29	0.93	0.003	0.001	37.66	0.003	104.39
**swing time (s)**									**swing time (s)**								
0.05	0.85	0.76	0.91	<0.001	0.026	7.82	0.071	21.67	0.05	0.71	0.24	0.91	0.002	0.021	5.60	0.057	15.54
0.25	0.77	0.62	0.86	<0.001	0.026	7.32	0.073	20.29	0.25	0.81	0.47	0.95	0.001	0.014	3.66	0.040	10.14
0.5	0.93	0.88	0.96	<0.001	0.012	3.11	0.033	8.63	0.5	0.84	0.53	0.96	<0.001	0.013	3.07	0.035	8.50
0.75	0.93	0.88	0.96	<0.001	0.010	2.55	0.029	7.08	0.75	0.82	0.49	0.95	0.001	0.016	3.83	0.045	10.61
0.95	0.87	0.79	0.93	<0.001	0.019	4.24	0.052	11.77	0.95	0.93	0.76	0.98	<0.001	0.013	2.81	0.035	7.78
max	0.85	0.76	0.91	<0.001	0.030	6.02	0.082	16.69	Max	0.88	0.62	0.97	<0.001	0.021	4.23	0.057	11.72
mean	0.91	0.85	0.95	<0.001	0.012	2.98	0.032	8.27	Mean	0.84	0.52	0.95	<0.001	0.012	2.97	0.034	8.22
min	0.72	0.56	0.83	<0.001	0.036	13.24	0.100	36.71	Min	0.85	0.55	0.96	<0.001	0.026	8.96	0.072	24.83
std	0.93	0.88	0.96	<0.001	0.009	21.42	0.024	59.37	Std	0.90	0.53	0.97	<0.001	0.007	22.58	0.020	62.60
CV	0.90	0.83	0.94	<0.001	0.030	27.72	0.083	76.84	CV	0.88	0.44	0.97	<0.001	0.018	23.40	0.050	64.86
variability	0.94	0.89	0.96	<0.001	0.008	21.60	0.022	59.87	variability	0.88	0.44	0.97	<0.001	0.005	18.06	0.013	50.06
asymmetry	0.79	0.67	0.88	<0.001	0.008	37.86	0.021	104.94	asymmetry	0.95	0.83	0.99	<0.001	0.007	33.07	0.020	91.67

**Table 4:** Reliability statistics for spatial and spatiotemporal gait characteristics for PwMS and HCs. Colour code **ICC**: Poor < 0.5, moderate = 0.5–0.75, good = 0.75–0.9, excellent > 0.9; **SEM%**: low ≤10%, High >10%. **MDC%**: low ≤20%, 20% < acceptable < 40%, high ≥40%.

PwMS									HCs								
	ICC (2,1)	(lb,	ub)	p	SEM	SEM (%)	MDC	MDC (%)		ICC (2,1)	(lb,	ub)	p	SEM	SEM (%)	MDC	MDC (%)
**Spatial**									**Spatial**								
**step length (m)**									**step length (m)**								
0.05	0.94	0.90	0.97	<0.001	0.041	8.94	0.114	24.79	0.05	0.45	-0.11	0.81	0.061	0.095	18.87	0.263	52.31
0.25	0.94	0.90	0.97	<0.001	0.032	5.99	0.090	16.60	0.25	0.70	0.23	0.91	0.006	0.046	7.73	0.128	21.44
0.5	0.94	0.89	0.96	<0.001	0.029	5.01	0.082	13.90	0.5	0.92	0.74	0.98	<0.001	0.022	3.39	0.061	9.40
0.75	0.93	0.87	0.96	<0.001	0.030	4.73	0.083	13.12	0.75	0.92	0.73	0.98	<0.001	0.025	3.63	0.068	10.07
0.95	0.78	0.65	0.87	<0.001	0.059	8.58	0.163	23.77	0.95	0.96	0.80	0.99	<0.001	0.020	2.74	0.054	7.58
max	0.53	0.30	0.70	<0.001	0.155	19.68	0.430	54.56	max	0.96	0.86	0.99	<0.001	0.022	2.81	0.061	7.78
mean	0.95	0.91	0.97	<0.001	0.028	4.75	0.077	13.16	mean	0.90	0.67	0.97	<0.001	0.025	3.92	0.068	10.87
min	0.75	0.60	0.85	<0.001	0.072	26.99	0.200	74.80	min	0.63	0.07	0.89	0.005	0.064	23.01	0.178	63.79
std	0.84	0.73	0.90	<0.001	0.020	24.02	0.054	66.59	std	0.45	−0.08	0.81	0.048	0.035	43.93	0.097	121.77
CV	0.93	0.87	0.96	<0.001	0.030	19.18	0.082	53.15	CV	0.33	−0.19	0.75	0.115	0.066	51.28	0.183	142.15
variability	0.78	0.65	0.87	<0.001	0.021	28.78	0.058	79.78	variability	0.59	0.07	0.87	0.014	0.023	32.15	0.063	89.12
asymmetry	0.78	0.64	0.87	<0.001	0.024	53.39	0.067	148.00	asymmetry	0.70	0.20	0.91	0.007	0.023	59.55	0.063	165.07
**stride length (m)**									**stride length (m)**								
0.05	0.93	0.88	0.96	<0.001	0.076	7.57	0.212	20.99	0.05	0.68	0.19	0.90	0.008	0.092	8.22	0.255	22.79
0.25	0.95	0.91	0.97	<0.001	0.059	5.31	0.165	14.73	0.25	0.81	0.47	0.94	0.001	0.069	5.71	0.192	15.83
0.5	0.94	0.89	0.96	<0.001	0.059	5.01	0.163	13.88	0.5	0.92	0.72	0.98	<0.001	0.044	3.49	0.123	9.69
0.75	0.89	0.82	0.94	<0.001	0.073	5.91	0.201	16.39	0.75	0.96	0.86	0.99	<0.001	0.033	2.49	0.091	6.89
0.95	0.89	0.82	0.94	<0.001	0.069	5.36	0.192	14.85	0.95	0.95	0.83	0.99	<0.001	0.038	2.77	0.106	7.69
max	0.84	0.74	0.91	<0.001	0.093	6.77	0.259	18.76	max	0.95	0.82	0.98	<0.001	0.042	2.91	0.116	8.08
mean	0.94	0.90	0.97	<0.001	0.056	4.80	0.155	13.32	mean	0.91	0.69	0.97	<0.001	0.048	3.81	0.133	10.55
min	0.84	0.74	0.91	<0.001	0.111	13.45	0.308	37.28	min	0.81	0.44	0.95	<0.001	0.058	6.22	0.161	17.25
std	0.74	0.59	0.85	<0.001	0.027	26.30	0.074	72.90	std	0.70	0.25	0.91	0.004	0.024	25.30	0.067	70.12
CV	0.82	0.71	0.90	<0.001	0.031	30.78	0.085	85.31	CV	0.56	0.02	0.85	0.025	0.025	32.02	0.069	88.75
**Spatiotemporal**									**Spatiotemporal**								
**step velocity (m/s)**									**step velocity (m/s)**								
0.05	0.92	0.87	0.96	<0.001	0.090	10.37	0.250	28.73	0.05	0.63	0.12	0.88	0.012	0.155	16.70	0.429	46.30
0.25	0.94	0.90	0.97	<0.001	0.064	6.16	0.177	17.07	0.25	0.88	0.63	0.97	<0.001	0.064	5.76	0.177	15.96
0.5	0.95	0.91	0.97	<0.001	0.055	4.84	0.152	13.41	0.5	0.95	0.81	0.99	<0.001	0.037	3.06	0.102	8.49
0.75	0.92	0.86	0.95	<0.001	0.065	5.32	0.181	14.73	0.75	0.93	0.76	0.98	<0.001	0.045	3.57	0.126	9.88
0.95	0.82	0.70	0.89	<0.001	0.092	6.74	0.255	18.69	0.95	0.93	0.73	0.98	<0.001	0.050	3.67	0.139	10.17
max	0.53	0.30	0.70	<0.001	0.249	15.49	0.691	42.94	max	0.75	0.31	0.93	0.003	0.166	10.26	0.460	28.45
mean	0.95	0.91	0.97	<0.001	0.054	4.79	0.150	13.28	mean	0.93	0.78	0.98	<0.001	0.045	3.79	0.125	10.52
min	0.80	0.67	0.88	<0.001	0.124	24.54	0.343	68.02	min	0.66	0.14	0.90	0.004	0.106	19.88	0.293	55.10
std	0.76	0.62	0.86	<0.001	0.045	25.86	0.124	71.68	std	0.50	−0.03	0.83	0.030	0.053	32.92	0.146	91.25
CV	0.89	0.81	0.93	<0.001	0.039	22.98	0.108	63.70	CV	0.40	−0.15	0.78	0.081	0.054	38.20	0.149	105.89
variability	0.75	0.60	0.85	<0.001	0.045	27.85	0.125	77.19	variability	0.69	0.19	0.91	0.002	0.037	24.93	0.102	69.11
asymmetry	0.73	0.57	0.84	<0.001	0.036	44.41	0.100	123.09	asymmetry	0.61	0.05	0.88	0.020	0.025	41.49	0.068	115.01
**stride velocity (m/s)**									**stride velocity (m/s)**								
0.05	0.93	0.88	0.96	<0.001	0.077	8.05	0.213	22.31	0.05	0.85	0.54	0.96	<0.001	0.074	7.19	0.204	19.94
0.25	0.95	0.91	0.97	<0.001	0.060	5.65	0.167	15.65	0.25	0.92	0.73	0.98	<0.001	0.050	4.45	0.139	12.34
0.5	0.96	0.93	0.98	<0.001	0.050	4.45	0.139	12.33	0.5	0.95	0.83	0.99	<0.001	0.040	3.35	0.111	9.30
0.75	0.94	0.90	0.97	<0.001	0.055	4.64	0.153	12.87	0.75	0.96	0.85	0.99	<0.001	0.038	3.05	0.105	8.45
0.95	0.94	0.90	0.97	<0.001	0.053	4.16	0.146	11.53	0.95	0.95	0.84	0.99	<0.001	0.042	3.22	0.116	8.93
max	0.85	0.76	0.91	<0.001	0.091	6.69	0.254	18.56	max	0.85	0.54	0.96	<0.001	0.081	5.91	0.226	16.39
mean	0.95	0.92	0.97	<0.001	0.054	4.76	0.148	13.19	mean	0.94	0.81	0.98	<0.001	0.041	3.50	0.114	9.71
min	0.82	0.71	0.89	<0.001	0.114	14.59	0.317	40.44	min	0.88	0.60	0.97	<0.001	0.049	5.70	0.135	15.80
std	0.67	0.48	0.80	<0.001	0.030	27.37	0.083	75.87	std	0.61	0.10	0.88	0.012	0.024	23.51	0.066	65.18
CV	0.85	0.75	0.91	<0.001	0.029	26.12	0.080	72.41	CV	0.60	0.08	0.87	0.017	0.024	27.23	0.067	75.48
